# The concurrence of an enterocutaneous fistula and granulomatosis with polyangiitis: The role of immunosuppression as a bridge to definitive surgical treatment

**Published:** 2021-11-06

**Authors:** Imad Hachem, Roy Hajjar, Frank Schwenter, Jean-Richard Goulet, Herawaty Sebajang

**Affiliations:** ^1^Digestive Surgery Service, Centre Hospitalier de l’Université de Montréal, Montreal, Canada; ^2^Faculty of Medicine, Université Laval, Quebec City, Canada; ^3^Department of Surgery, Faculty of Medicine, Université de Montréal, Montreal, Canada; ^4^Department of Medicine, Centre Hospitalier de l’Université de Montréal, Montreal, Canada; ^5^Department of Medicine, Faculty of medicine, Université de Montréal, Montreal, Canada

**Keywords:** granulomatosis with polyangiitis, enterocutaneous fistula, immunosuppression in sepsis, surgical treatment

## Abstract

**Background and Aim:**

Granulomatosis with polyangiitis (GPA) is a systemic disease that consists of vasculitis and granulomatous inflammation, and that usually affects the respiratory tract, the ear, nose, and throat sphere, and the kidneys. GPA may also cause skin manifestations that include ulcerations, nodules, or papules. An enterocutaneous fistula (ECF) is an abnormal tract that connects the skin surface to the gastrointestinal system.

**Methods:**

We report the first case of an ECF as a concurrent clinical manifestation during a new-onset GPA in a 68-year-old male patient.

**Results:**

The patient presented with an abdominal cutaneous wound with subcutaneous abscess that evolved into an ECF with spontaneous enteric drainage. He also complained of nasal crusting, epistaxis, and cough, with further investigation revealing bilateral pulmonary nodules. Transthoracic biopsy was performed and was suggestive of necrotizing vasculitis. A diagnosis of autoimmune vasculitis was highly suspected, and an immunosuppressive regimen of corticosteroid and intravenous cyclophosphamide was initiated. Significant improvement was noted in nasal manifestations, cough, and the output of the ECF. Definitive surgical management of the ECF was performed successfully.

**Conclusion:**

To the best of our knowledge, the presentation of a GPA with an ECF has not been previously reported and poses major challenges to medical and surgical treatment, as it constitutes a dilemma as to how to address an autoimmune process requiring immunosuppression in the context of an infectious condition. This presentation suggests that immunosuppression in these patients may still be considered.

**Relevance for Patients:**

The concomitant presence of an ECF with abscess, an infectious process, and of an autoimmune disorder requiring immunosuppression is a major medical challenge. This case suggests that immunosuppression may still be considered in these patients to promote a better control of the concomitant ECF before definitive surgical therapy.

## 1. Introduction

Granulomatosis with polyangiitis (GPA), previously known as Wegener’s granulomatosis, is a rare systemic disease that consists of systemic vasculitis and granulomatous inflammation [1]. It could affect any age group but is usually diagnosed between the ages of 40 and 65 years [2]. Conventionally, the presenting symptoms usually involve the upper respiratory tract, the ear, nose, and throat (ENT) sphere, the lungs, and the kidneys [1]. Inflammatory lesions may affect other systems including the nervous system, the heart, the gastrointestinal tract, and the skin [1,3,4]. Microscopically, the disease is characterized by granulomatous changes, necrosis, and vasculitis that usually affect small- to medium-sized vessels [1,5]. Cutaneous manifestations of GPA may be present in 25-50% of patients and usually consist of ulcerations, nodules, papules, or purpura [1,3,6]. GPA may be fatal and requires a prompt treatment, which is usually based on systemic immunosuppression [1,7]. We report an enterocutaneous fistula (ECF) – or a tract connecting the skin surface to the gastrointestinal system [8] – as a concomitant clinical manifestation during a new-onset GPA. To the best of our knowledge, this presentation has not been previously reported.

## 2. Case Presentation

A 68-year-old male patient was transferred to our referral center for an abdominal ECF at the site of a mesh from a previous hernia repair. His past medical history includes coronary artery disease with coronary bypass surgery 12 years before the present events, a ventral hernia repair with mesh 22 years before the current episode, and a smoking history of approximately 3-4 pack-years.

**Figure 1:**
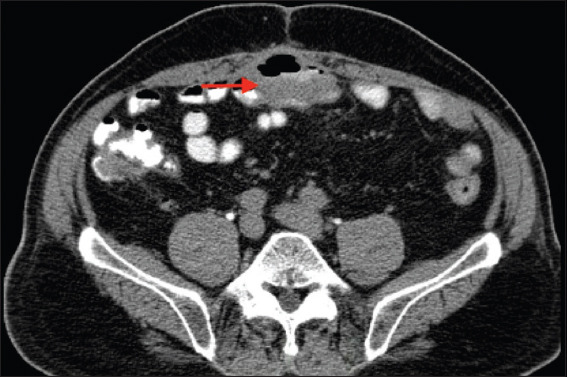
Abdominopelvic computed tomography scan displaying an anterior abscess (red arrow) at the site of a previous hernia repair with synthetic mesh.

**Figure 2:**
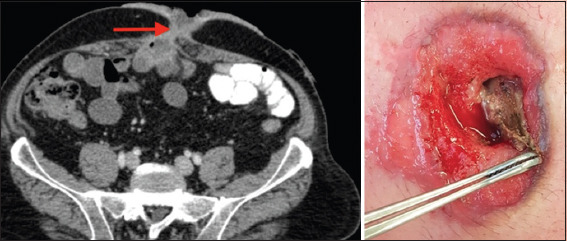
Abdominopelvic computed tomography scan (left) displaying an anterior enterocutaneous fistula (red arrow) at the site of a previous hernia repair with synthetic mesh. Photo of the fistula (right) with the exposed mesh.

**Figure 3:**
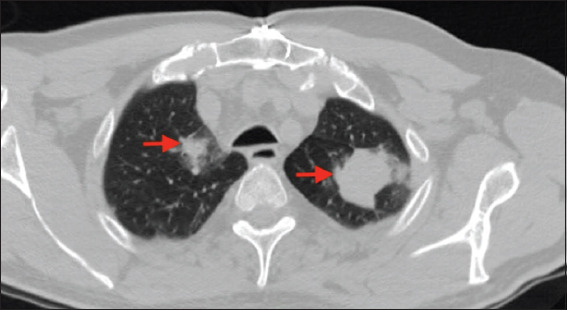
Thoracic computed tomography scan displaying bilateral pulmonary nodules (red arrows)

The patient has been complaining of a superficial non-healing abdominal wound lasting for almost a year, which led him to consult at another hospital due to increased pain, local erythema, and fever. Imaging revealed the presence of a subcutaneous abscess (Figure 1), for which large spectrum antibiotic therapy was initiated. After 2 weeks of intravenous antibiotherapy, the abscess did not resolve and enteric content started leaking from a skin opening, suggesting the progression to an ECF (Figure 2). Due to subacute mild non-productive cough, the patient underwent as well a thoracic computed tomography (CT) scan that displayed bilateral cavitary nodules (Figure 3) which seemed to progress in size on a second thoracic CT scan performed a month later. The patient’s daily activities and occupation could not be linked to an increased risk of pulmonary infectious agents or inhalation injury, and he had no previous history of tuberculosis. Moreover, the patient complained of nasal crusting with epistaxis. Due to the complexity of the presentation, the patient was transferred to our university hospital for multidisciplinary management.

An infectious or a neoplastic process was suspected. Extensive microbiological investigations failed to identify a potential etiological agent at the ECF site or in the pulmonary lesions. Culture of the abdominal subcutaneous abscess revealed the expected presence of enteric pathogens, including *Enterococcus sp*., *Enterobacter cloacae*, *Escherichia coli*, and multiple species of *Candida*. No bacterial or mycotic agents were identified on bronchoalveolar lavage. The patient was thus maintained on total parenteral nutrition and on a large spectrum regimen of intravenous antibiotic therapy, consisting initially of piperacillin-tazobactam, then imipenem/cilastatin, vancomycin, and fluconazole, followed by ciprofloxacin and metronidazole and fluconazole. Antibiotic agents were based on antibiogram susceptibility tests and lasted for several weeks with no noticeable clinical improvement. The white blood count was normal since his arrival to our center (<11.0×10^9^/L), but the C-reactive protein (CRP) remained high (>100 mg/L) and the patient had a persistent fever despite maximal antibiotic treatment. To rule out a neoplastic process, a surgical biopsy of the ECF opening was performed and revealed cutaneous ulcerations and inflammatory granulation tissue, while transbronchial lung biopsy revealed pulmonary inflammatory changes without dysplasia. The output of the fistula was estimated at approximately 2-3 liters/24 h and responded only slightly to octreotide and loperamide.

To confirm the etiology of the pulmonary nodules, a subsequent transthoracic biopsy was performed and revealed the presence of necrotizing damage affecting intraparenchymatous arterioles and venules, suggestive of a necrotizing vasculitis. Complementary blood analyses revealed positive proteinase 3 anti-neutrophil cytoplasmic antibody (PR3-ANCA/c-ANCA) at >8.0 antibody index and negative myeloperoxidase ANCA perinuclear p-ANCA. With negative infectious and neoplastic investigations, a diagnosis of an autoimmune disorder, specifically GPA, was highly suspected, which could have also been the cause of epistaxis and nasal crusting. No decline in kidney function, suggestive of renal damage, was noted.

With a high probability of an autoimmune vasculitis, and with the lack of improvement with antibiotic therapy, an immunosuppressive regimen of corticosteroid and intravenous pulse cyclophosphamide was initiated. On the initiation of this treatment, significant improvement was noted with a decrease in nasal manifestations and cough. Approximately 2 weeks after the initiation of the immunosuppressive treatment, a significant decrease in the size of the pulmonary lesions was noted, and antibiotic therapy was discontinued. Furthermore, the ECF output decreased significantly to <1 liter/24 h, and the levels of CRP normalized as well.

**Figure 4:**
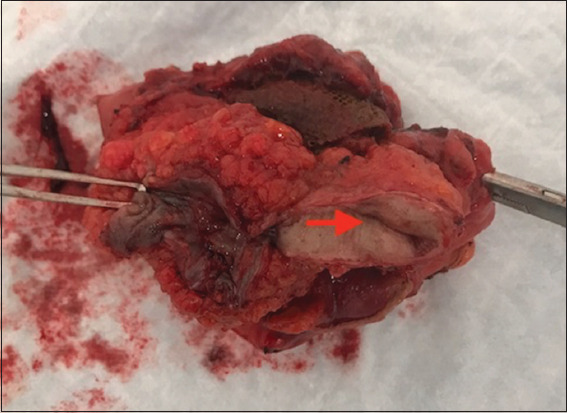
Surgical specimen of the resected enterocutaneous fistula and the synthetic mesh, with depiction of the healing fistula opening (red arrow).

After 2 months of immunosuppressive treatment, the patient underwent surgical excision of the ECF (Figure 4) with small bowel resection and anastomosis. He underwent an open cholecystectomy as well for a concomitant calculous cholecystitis, which was complicated by biliary trauma that further warranted a Roux-en-Y hepaticojejunostomy. After surgery, he developed an anastomotic leak that was controlled with intra-abdominal drainage and antibiotic therapy.

The patient was discharged after a hospital stay of 143 days, with close outpatient follow-up and treatment with azathioprine and decreasing doses of prednisone. No signs of ECF recurrence were noted 1 year after the episode of care.

## 3. Discussion

We hereby present the first reported case of GPA presenting with an ECF with infected mesh that has responded favorably to immunosuppressive medication before surgery.

An ECF is a non-physiological connection between the bowel lumen and the skin epithelium that result in external leaking of bowel content [7,8]. This condition is associated with high morbidity and mortality rates of up to 33% [7]. The latter seems to be mainly related to the associated sepsis [7]. It has been reported that ECFs arise after an abdominal surgical procedure in up to 85% of the cases, with the remaining cases being due to inflammatory or neoplastic conditions [7,9]. The mainstay of treatment consists of nutritional support and the control of the fistula’s output and sepsis [7,10], which leads to spontaneous closure in up to 92% of patients [10].

In the present case, the patient had no objectifiable cause for the new-onset ECF except a previous ventral hernia repair 22 years ago which included the use of a synthetic mesh. Although late ECF due to the erosion of the underlying bowel by the mesh has been previously reported [11], the patient’s presentation coincided with respiratory and nasal symptoms rather suggestive of a systemic inflammatory process. Despite no histological evidence of vasculitis at the fistula site, GPA is known to present as cutaneous lesions in 10% of the cases [3], and these lesions may usually range from papules to more severe ulcerations [1,3]. The GPA diagnosis was confirmed by pulmonary biopsy that displayed typical signs of necrotizing vasculitis [5], as well as the elevation of c-ANCA/PR3. The significant improvement of the ECF with immunosuppressive treatment is highly suggestive of an association between the fistula and the systemic inflammatory process. However, whether the ECF is a manifestation of the autoimmune process, or a triggering factor cannot be confirmed beyond any doubt. It is known that the pathophysiology of GPA remains poorly understood, but it is believed that infectious processes, in addition to genetic predisposition and other environmental factors, constitute potential risk factors for this disease [2]. In this case, it is worth noting as well that ANCA may be elevated in other conditions such as infectious conditions, warranting, therefore, other investigations to confirm the diagnosis of an autoimmune disorder such as a GPA [12].

As previously mentioned, ECFs with an underlying abscess pose major challenges in terms of treatment, and an additional immunosuppression may be hazardous in such a context. This regimen was, however, required to improve the patient’s pulmonary condition to allow for an abdominal surgical intervention. Interestingly, the ECF site displayed accelerated wound healing and a considerable decrease of the fistula output when corticosteroids and cyclophosphamide were initiated. This may suggest that the inflammatory process at the fistula site may have been potentially exacerbated by the autoimmune process, leading to a good response to systemic immunosuppression. This observation remains nonetheless hypothetical as the specific triggering factor for the ECF as this specific time cannot be confirmed.

Nonetheless, it remains important to note that immunosuppression in patients with sepsis is perilous and requires a high level of suspicion of a non-infectious etiology, in addition to significant expected benefits, to justify its use.

## 4. Conclusion

GPA is a systemic disease that typically induces vasculitis of small- and medium-sized vessels, which is typically associated with lesions in the ENT sphere, pulmonary tract, and kidneys, and less commonly with cutaneous inflammatory lesions [3,4,6]. The concomitant presence of an ECF with a systemic presentation of GPA has not been previously described. This rare occurrence poses major challenges to medical and surgical management, as it constitutes a dilemma as to how to address an autoimmune process requiring immunosuppression in the context of an infectious condition. We present the first case where immunosuppressive management led to an improvement of a persistent ECF and allowed a successful subsequent surgical intervention. More cases are, however, required to validate the safety of this approach.

### Informed Consent

The patient has provided written consent for the publication of the present article.

### Conflicts of Interest

The authors declare no conflicts of interest.
